# In your face: facial metrics predict aggressive behaviour in the laboratory and in varsity and professional hockey players

**DOI:** 10.1098/rspb.2008.0873

**Published:** 2008-08-19

**Authors:** Justin M Carré, Cheryl M McCormick

**Affiliations:** 1 Department of Psychology, Brock University500 Glenridge Avenue, St. Catharines, Ont., Canada L2S 3A1; 2 Centre for Neuroscience, Brock University500 Glenridge Avenue, St Catharines, Ont., Canada L2S 3A1

**Keywords:** aggression, sex differences, dominance, sexual selection, face

## Abstract

Facial characteristics are an important basis for judgements about gender, emotion, personality, motivational states and behavioural dispositions. Based on a recent finding of a sexual dimorphism in facial metrics that is independent of body size, we conducted three studies to examine the extent to which individual differences in the facial width-to-height ratio were associated with trait dominance (using a questionnaire) and aggression during a behavioural task and in a naturalistic setting (varsity and professional ice hockey). In study 1, men had a larger facial width-to-height ratio, higher scores of trait dominance, and were more reactively aggressive compared with women. Individual differences in the facial width-to-height ratio predicted reactive aggression in men, but not in women (predicted 15% of variance). In studies 2 (male varsity hockey players) and 3 (male professional hockey players), individual differences in the facial width-to-height ratio were positively related to aggressive behaviour as measured by the number of penalty minutes per game obtained over a season (predicted 29 and 9% of the variance, respectively). Together, these findings suggest that the sexually dimorphic facial width-to-height ratio may be an ‘honest signal’ of propensity for aggressive behaviour.

## 1. Introduction

Most studies that have investigated facial characteristics and sexual selection have focused on what is perceived as attractive to an observer (Rhodes 2006). Some evidence suggests that certain facial judgements may reflect inherent processing mechanisms in that newborns' preferences for faces parallel those of adults (e.g. Langlois *et al*. 1987; Slater *et al*. 2000). Although judgements of attractiveness also are influenced by experience (e.g. Peskin & Newell 2004), perception of attractiveness is thought be part of human evolutionary heritage, perhaps as an honest signal of health (Thornhill & Gangestad 1999). Sexual dimorphism in the face is one such signal that may have been shaped by intra- and intersexual selection (Little *et al*. 2008). For instance, men with masculine facial features may have obtained increased access to valued resources (i.e. resources important for survival and reproduction) because they are regarded as socially and physically dominant by their rivals (Mueller & Mazur 1996; Swaddle & Reierson 2002). Also, facial masculinity is generally found attractive in men, perhaps serving as an honest signal of health (Rhodes 2006; Rhodes *et al*. 2007). Further, a recent study found a positive correlation between salivary testosterone concentrations and ratings of facial masculinity (Penton-Voak & Chen 2004). The immunocompetence handicap hypothesis posits that testosterone is responsible for the development of male secondary sex traits (e.g. facial masculinity), but it also has a negative impact on the immune system (Folstad & Karter 1992). Therefore, only high-quality (i.e. healthy, good ‘genes’) men can afford to display these characteristics without suffering the costs of parasite loads (Folstad & Karter 1992).

In addition to providing information as to the personality and health of a target, some recent studies suggest that characteristics of the face may also provide cues as to the behavioural tendencies of the target. For example, women's judgements of the extent to which a man was interested in infants based on his face predicted his actual interest in infants (as measured in a laboratory task; Roney *et al*. 2006). Raters' judgements of facial masculinity (Rhodes *et al*. 2005) and dominance (Mazur *et al*. 1994) predicted sexual behaviour of men. People also show some accuracy at identifying ‘cheaters’ in a Prisoner's Dilemma game based on facial photographs (Verplaetse *et al*. 2007). Together, these findings suggest that people can make accurate inferences about others' personality traits and behavioural dispositions based on certain signals conveyed by the face. The precise facial metrics used to make these trait judgements are not well understood (Danel & Pawlowski 2007). Recently, Weston *et al*. (2007) described the facial width-to-height ratio, a sexual dimorphism in the structure of the face that was independent of body size, from a morphometric analysis of an ontogenetic series of skulls. In brief, males and females were found to have different growth trajectories that diverge at puberty for bizygomatic width and not for upper facial height, leading to a width-to-height facial dimorphism (greater ratio in men than in women) that is independent of increased body size. The sex difference in the facial width-to-height ratio emerged around puberty, which is when sex differences in facial structure related to body size appear, in part due to increased testosterone concentrations at puberty in boys (Verdonck *et al*. 1999). Thus, the sexual dimorphism in facial width-to-height may reflect a sexual selection pressure that is independent of selection for body size.

Here, we conducted three studies to examine the extent to which the face width-to-height ratio predicted dominance and aggressive behaviour. In study 1, we first examined whether the facial width-to-height sexual dimorphism, previously described in skulls (Weston *et al*. 2007), can be found in the photographs of faces. We also investigated the extent to which within-sex variability in the width-to-height ratio in the upper face was associated with within-sex variability in a sexually dimorphic personality trait, dominance, and in behavioural aggression. Dominance and aggression were chosen as measures because facial signals of dominance and/or aggression may not only influence mate preference (intersexual selection), but may also be important signals moderating intermale behaviour (intrasexual selection). For studies 2 and 3, we examined whether a relationship between individual differences in the facial width-to-height ratio would predict aggressive behaviour outside of a laboratory setting. The association between facial width-to-height ratio and aggressive behaviour (defined as the number of penalty minutes obtained per game) was tested in male varsity hockey players (study 2) and in professional ice hockey (Canadian teams in the National Hockey League (NHL); study 3).

## 2. Material and methods

### (a) Participants

In study 1, 88 undergraduate students (37 men and 51 women; mean age=18.98 years, s.d.=1.15) took part for course credit and a $5 honorarium. Eighty-two per cent of the participants self-identified as Caucasian, with the remaining 18 per cent representing a diversity of ethnicities. All procedures of the study were approved by the university's ethical review committee.

### (b) Face ratios

In study 1, photographs were taken with a Nikon D50 digital camera. ImageJ (NIH open-source software) was used to measure the distance between the lip and brow (height of upper face) and the left and right zygion (bizygomatic width) of the digitized images, based on Weston *et al*. (2007; figure 1). Inter-rater reliability was high for all measures (distance between left and right zygion: *r*=0.996, *p*<0.001; distance between the lip and brow: *r*=0.989, *p*<0.001; width-to-height ratio: *r*=0.985, *p*<0.001).

For study 2, photographs were obtained from 21 undergraduate male varsity hockey players (mean age=22.81 years, s.d.=1.29) from the university's website (all players whose pictures were available except for goalkeepers because these individuals are typically not in a position to obtain penalties, the measure of aggression) and measured as in study 1. All the pictures were facing forward; however, some individuals did not have a neutral expression (i.e. some were smiling). All measurements had good inter-rater reliability (facial height: *r*=0.98, *p*<0.001; facial width: *r*=0.94, *p*<0.001; width-to-height ratio: *r*=0.90, *p*<0.001).

For study 3, photographs were obtained for every player who played on the Canadian teams of the NHL during the 2007–2008 season (Calgary Flames, Edmonton Oilers, Montreal Canadiens, Ottawa Senators, Toronto Maple Leafs and Vancouver Canucks) whose pictures were available on the Entertainment and Sports Programming Network (ESPN) website (*n*=126). Two pictures had to be excluded because the individuals were not facing forward and the tilt of the head would compromise the measurement of the facial width-to-height ratio. Further, we excluded photographs from goalkeepers (*n*=12) because these individuals are typically not in a position to obtain penalties (the measure of aggression). The final sample was *n*=112. Facial width-to-height ratios were calculated as in studies 1 and 2. All measurements had good inter-rater reliability (facial height: *r*=0.97, *p*<0.001; facial width: *r*=0.996, *p*<0.001; width-to-height ratio: *r*=0.96, *p*<0.001).

### (c) Trait dominance

Participants completed a 10-item questionnaire assessing trait dominance (International Personality Item Pool scales; Goldberg *et al*. 2006). Some examples of items include ‘Like having authority over others’ and ‘Want to be in charge’. Responses were scored on a Likert scale ranging from −2 (very inaccurate) to +2 (very accurate), and had high reliability (Cronbach's alpha=0.82).

### (d) Point Subtraction Aggression Paradigm

To measure aggressive behaviour, we used a modified version of the Point Subtraction Aggression Paradigm (PSAP; Cherek 1981). This measure is positively correlated with various self-report measures of aggression (Gerra *et al*. 2007; Golomb *et al*. 2007). In brief (see also Carré & McCormick 2008), participants were led to believe that they would be paired with a same-sex partner (in actuality, an E-Prime computer program) on a task that required them to select among three response options to earn points that would be exchangeable for money. Pressing response option no. 1 a hundred consecutive times would cause the point counter on the screen to enlarge, flash several times with positive signs around it, and increase the point counter total by one. It was explained to participants that the point counter might flash several times with negative signs around it, resulting in a one-point decrease in the point counter total. They were told that this meant that their partner (actually the computer program) had stolen a point, and each stolen point would be added to the partner's counter. Participants could respond by continuing to select option no. 1 (point reward) or could switch to option no. 2 or 3. Pressing option no. 2 ten times would steal a point from their partner; however, participants were instructed that they were randomly assigned to the experimental condition whereby they, unlike their partner, would not keep any points stolen. Pressing option no. 3 ten times would protect their point counter against theft of points for a brief time. Thus, the dependent variables from the PSAP measure were option nos. 1 (reward earned), 2 (aggression) and 3 (protection). Selection of option no. 2 was considered reactive aggression because the participants did not increase reward, and in fact lost opportunity to increase reward, each time option no. 2 was selected.

### (e) Study 1 procedure

Participants arrived in groups of two or four and first completed a demographic and trait dominance questionnaire. Next, participants were photographed while in a seated position and maintaining a neutral facial expression. Participants were escorted to separate rooms for the PSAP procedure. The PSAP took approximately 40 min to complete, after which they completed a brief questionnaire designed to assess whether they were aware of the deception used in the experiment. Responses confirmed that participants believed that they were playing against another person.

### (f) Aggression measure in studies 2 and 3

The penalty minutes that each player accrued per number of games played during the 2007–2008 season (obtained from the Ontario University Athletics website for study 2 and from ESPN's website for study 3) were used as the measure of aggression. Penalties included behaviours such as slashing, cross-checking, high-sticking, boarding, elbowing, checking from behind, fighting and so on. These behaviours meet the classic definition of aggressive behaviour as any act that is intended to harm another individual, who, in turn, is motivated to avoid the behaviour (Baron & Richardson 1994).

### (g) Statistics

Gender differences in trait dominance and facial width-to-height ratio were examined using multivariate analysis of variance (MANOVA), with follow-up *t*-tests. Multiple linear regression analysis was used to examine the relationship between predictor variables (trait dominance, facial width-to-height ratio and trait dominance by facial width-to-height ratio interaction) and aggressive behaviour as measured by the PSAP. The main assumptions underlying linear regression (e.g. outliers, linear relationship between predictors and criterion, multicollinearity, independence of observations, homoscedasticity and errors normally distributed) were examined and were all met. Also, Pearson's correlation coefficients were computed to examine the bivariate association between the facial width-to-height ratio and aggressive behaviour in varsity and professional ice hockey players. All analyses conducted were two-tailed and the level of significance was set at *p*<0.05.

## 3. Results

### (a) Study 1

Tests for gender differences in trait dominance, facial ratio and aggressive behaviour consisted of MANOVA and follow-up *t*-tests. There was a main effect of gender (*F*_5,82_=3.04, *p*=0.01): men had a greater facial ratio (*t*_86_=2.33, *p*=0.02, Cohen's *d*=0.50); scored higher on trait dominance (*t*_86_=2.15, *p*=0.04, Cohen's *d*=0.46); and were more aggressive than women (*t*_86_=2.18, *p*=0.03, Cohen's *d*=0.47). Men and women did not differ in reward (*t*_86_=−0.80, *p*=0.43, Cohen's *d*=0.18) or protection (*t*_86_=0.66, *p*=0.51, Cohen's *d*=0.15) responses (table 1).

Separate regression analyses for men and women were computed with trait dominance and face ratio as predictors of aggressive behaviour. For men, face ratio predicted 15 per cent of unique variance in aggressive behaviour (*R*^2^=0.18, *F*_2,34_=3.60, *p*=0.04; *t*_36_=2.50, *p*=0.02; figure 2), but trait dominance was not a significant predictor of aggression (*p*=0.27). Furthermore, the face ratio by trait dominance interaction was not significant (*R*_change_^2^=0.001, *F*_1,33_=0.04, *p*=0.84). For women, face ratio and trait dominance did not predict aggressive behaviour (*R*^2^=0.03, *F*_2,41_=0.66, *p*=0.52), nor did the interaction (*R*_change_^2^=0.003, *F*_1,40_=0.14, *p*=0.72).

### (b) Study 2

Individual differences in face ratio in male hockey players explained 29.2 per cent of the variance in penalty minutes per game played (*r*=0.54, *p*=0.01; figure 3).

### (c) Study 3

Individual differences in the face width-to-height explained a significant proportion of the variance in aggressive behaviour (*r*=0.30, *p*=0.005) in NHL hockey players. Separate correlation coefficients were also computed for each individual team (figure 4). All correlation coefficients were in the positive direction and ranged from 0.17 to 0.51.

## 4. Discussion

In sum, a sexually dimorphic width-to-height ratio (men>women) in the upper face was evident in the photographs of an unselected sample of undergraduates, and this ratio predicted aggression in men assessed in a validated laboratory behavioural task and in a naturalistic setting (varsity and professional ice hockey players). Weston *et al*. (2007) first reported the sexually dimorphic facial width-to-height ratio in an analysis of a series of human skulls representing different stages of ontogeny. They found that the sex difference emerged around puberty, which is when sex differences in facial structure related to body size appear, in part due to increased testosterone concentrations at puberty in boys (Verdonck *et al*. 1999). This finding is consistent with a recent study demonstrating a positive correlation between salivary testosterone concentrations and ratings of facial masculinity (Penton-Voak & Chen 2004). The sex difference in the facial ratio observed in skulls was independent of body size and of other size-related facial variation, and thus suggests that this sexual dimorphism may reflect a selection pressure that is independent of body size (Weston *et al*. 2007). A similar sexual dimorphism in the face, which was independent of body size, was also reported in chimpanzees (Weston *et al*. 2004).

The data obtained here suggest that for men variation in the width-to-height ratio from neutral faces may be an honest signal of propensity for aggressive behaviour. Clearly, an angry facial expression is a direct way to communicate one's emotional state and behavioural intent. However, it remains possible that subtle cues from a neutral face may have been selected because they provide information as to an individual's behavioural dispositions. Notably, angry facial expressions consist of lowering the brow and raising the upper lip, a pattern of muscle activity that increases the facial width-to-height ratio. From an evolutionary perspective, these findings suggest that selection pressures may have shaped the perceptual system to be especially attuned to cues of threat and/or aggression. However, it will be important to examine the extent to which people are sensitive to individual differences in facial width-to-height ratio and whether this facial metric is used to guide behaviour. Another possibility is that the relationship observed between the facial metric and aggressive behaviour was partly influenced by the posture of the head in the photographs (e.g. more aggressive men may tilt their head upwards and thereby foreshorten the vertical measurement of the face). It is also possible that stronger relationships would have been observed between the facial ratio and aggressive behaviour if direct measurements of the face were made instead of using photographs and had we been able to control facial expression in the faces of the hockey players.

There was no relationship between trait dominance and aggressive behaviour in our sample, although individual differences in trait dominance were associated with self-report measures of trait aggression in both men and women in other studies (Archer & Webb 2006; Johnson *et al*. 2007). This disparity may reflect that the PSAP is a behavioural measure designed to assess situation-specific reactive aggression, whereas the other studies measured a broader range of aggression (physical, verbal, hostile and anger) across several situations using self-report. However, the higher trait dominance in men than in women found here is consistent with previous studies using similar self-report measures (Budaev 1999; Costa *et al*. 2001). Furthermore, the sex difference in aggressive behaviour on the PSAP is consistent with that reported in the literature (reviewed in Archer 2004). The fact that there were no sex differences in reward or protection responding on the PSAP suggests that men were equally motivated to earn reward and avoid punishment (i.e. point subtractions).

There is much research literature addressing the role of the face in social interactions, and there is some literature indicating that faces can be used to gauge certain personality traits above chance (e.g. Penton-Voak *et al*. 2006). Such judgements are made in less than 40 ms, made with high consistency and have some predictive values (Bar *et al*. 2006; Willis & Todorov 2006; Ballew & Todorov 2007). For example, women's judgements of the extent to which a man was interested in infants based on his face predicted his actual interest in infants (Roney *et al*. 2006). Judgements of competence, intelligence and leadership based on only the facial appearance of political candidates (and independent of age and attractiveness) predicted the outcome of the elections (Todorov *et al*. 2005). Judgements of dominance predicted career success (Mueller & Mazur 1996) and age at first copulation (Mazur *et al*. 1994). Another study reported that participants' judgements of the personality traits of power (competence, dominance and facial maturity) and warmth (likeability and trustworthiness) in the faces of CEOs of Fortune 500 companies predicted the profits of the CEO's company (Rule & Ambady 2008). However, whether the actual success of the individuals whose faces were judged is partly due to shared societal stereotypes (e.g. the success of attractive people is in part because they are judged as more intelligent; Zebrowitz *et al*. 2002) continues to be debated.

The novel finding of the present study is that individual differences in facial characteristics predict behaviour: Variability in a sexually dimorphic facial metric in men, which is independent of body size, predicted aggressive behaviour in a laboratory setting and in male hockey players. The relationship between within-sex variation in facial width-to-height and aggressive behaviour might reflect a common relationship to a third variable, such as organizational effects of testosterone as part of sexual differentiation in adolescence, which influences both the development of the physique and the nervous system (reviewed in Sisk & Zehr 2005; McCormick & Mathews 2007). The relationship between facial morphology and aggressive behaviour suggests that this characteristic may be an honest signal, perhaps comparable to honest signals in other species that predict factors such as phenotypic quality (Vanpe *et al*. 2007) or aggressive intent (Morestz & Morris 2006; Laidre & Vehrencamp 2008).

## Figures and Tables

**Figure 1:**
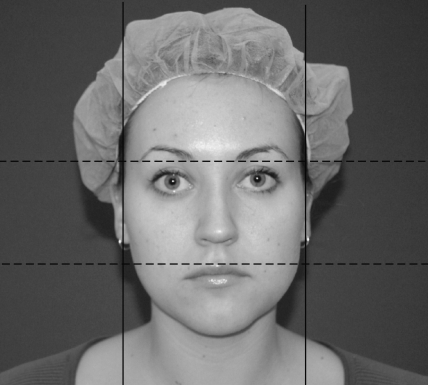
An example of the measures used for facial width-to-height ratio. Vertical lines represent the distance between the left and the right zygion (bizygomatic width). Horizontal lines represent the distance between the upper lip and brow (upper facial height).

**Figure 2:**
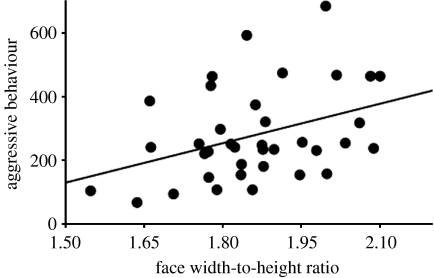
Scatter plot depicting the relationship between face width-to-height ratio and aggressive behaviour in undergraduate men (*n*=37, *r*=0.38 and *p*=0.02).

**Figure 3:**
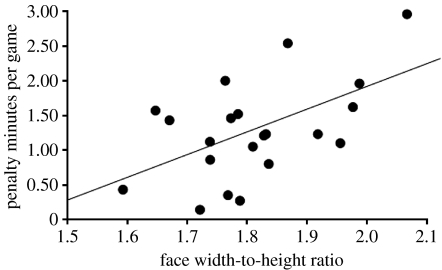
Scatter plot depicting the relationship between face width-to-height ratio and aggressive behaviour (penalty minutes per game) in male varsity hockey players (*n*=21, *r*=0.54 and *p*=0.01).

**Figure 4:**
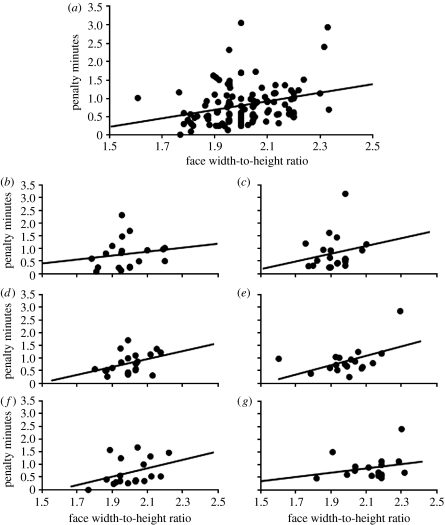
Scatter plots depicting the relationship between face width-to-height ratio and aggressive behaviour (number of penalty minutes per game played) in male professional hockey players for the six Canadian teams in the NHL (*n*=112) as a group (*a*) and for each individual team (*b*–*g*): (*a*) all Canadian NHL teams (*r*=0.30), (*b*) Calgary Flames (*r*=0.17), (*c*) Edmonton Oilers (*r*=0.20), (*d*) Montreal Canadiens (*r*=0.39), (*e*) Ottawa Senators (*r*=0.51), (*f*) Toronto Maple Leafs (*r*=0.37) and (*g*) Vancouver Canucks (*r*=0.24).

**Table 1:** Mean (s.e.m.) for women (*n*=51) and men (*n*=37) for each of the variables measured in study 1.

	women	men	*t* (d.f.=86)	*p*-value
face ratio	1.80 (0.10)	1.86 (0.13)	2.33	0.02
trait dominance	5.8 (5.5)	8.35 (5.5)	2.15	0.04
PSAP responses				
aggression	209 (151)	278 (145)	2.18	0.03
reward	2486 (406)	2423 (302)	−0.80	0.43
protection	294 (164)	316 (127)	0.66	0.51
